# Asthma prevalence and risk factors among children and adolescents living around an industrial area: a cross-sectional study

**DOI:** 10.1186/1471-2458-13-1038

**Published:** 2013-11-04

**Authors:** Giancarlo Ripabelli, Manuela Tamburro, Michela Lucia Sammarco, Guglielmo de Laurentiis, Andrea Bianco

**Affiliations:** 1 Chair of Hygiene, Department of Medicine and of Health Sciences, University of Molise, Via De Sanctis, Campobasso, 86100, Italy; 2 Chair of Respiratory Diseases, Department of Medicine and of Health Sciences, University of Molise, Campobasso, Italy

**Keywords:** Childhood asthma, Industrial area, Prevalence, Risk factors, Drug prescription

## Abstract

**Background:**

The exposure to air pollution has negative effects on human health, increasing the risk of respiratory diseases, such as asthma. Few data are yet available on the epidemiology of childhood asthma in some areas of Italy. The aim of the study was to estimate asthma prevalence and related risk factors in children and adolescents residents around the industrial area of Termoli, Molise region, Central-South Italy.

**Methods:**

Prevalence was assessed through the administration of modified ISAAC questionnaires filled out by parents of 89 children and adolescents for the identification of confirmed and probable cases, and by analyzing pediatricians’ databases on drug prescriptions for symptoms control and treatment of assisted population in the study area (n = 1,004), compared to a control area (n = 920) with lower industrialization. The association of asthma with risk factors was evaluated by univariate (Chi-square or Fisher’s Exact test) and regression logistic analysis.

**Results:**

A total of 22 (24.7%) asthmatics were identified, including both confirmed (n = 7; 7.9%) and probable cases (n = 15; 16.8%), most of them (n = 17; 77.3%) resident of Termoli town. All asthma cases were georeferenced based on the residence, however clusters were not found. Using drug prescriptions analysis, a higher prevalence (n = 138; 13.7%) of diagnosed cases was found. Lifetime history of both atopic dermatitis and bronchitis were significantly relateds to asthma cases, as well as an elevated body mass index, whose association is consistent with prevalence data of overweight/obese children living in the study area. Moreover, being resident of the town of Termoli was associated to the occurrence of cases.

**Conclusions:**

Although our data indicated a prevalence concordance with previous national studies in pediatric population, a definitive correlation with environmental industrial factors present in the study area was not established. However, asthma outcome was significantly associated to individuals living in the town of Termoli that, despite the industrial/manufacturing activities, is also subjected to a higher environmental pressure due to the presence of toll road, state highway, railroad, and seaport which may cause air pollution from motor vehicle traffic and increase asthma induction. This study provides hitherto unavailable data on asthma in childhood population living in an industrialized area which was never investigated before, could be part of a systematic review or meta-analysis procedure, might suggest significant findings for larger observational studies, and contribute to complete the frame of disease epidemiology in Italy.

## Background

Asthma is a chronic respiratory disease, characterized by episodes or attacks of impaired breathing, affecting up to 10% of adults and 30% of children [1,2]. Symptoms are caused by inflammation of small airways and may include bronchial hyperresponsiveness, recurrent attacks of wheezing, shortness of breath, chest tightness and coughing, particularly at night or early morning. The variable airflow obstruction is often reversible, either spontaneously or by treatment with bronchodilators or corticosteroids [3,4].

Onset of asthma usually begins early in life as a pattern of atopic wheezing that is exacerbated by allergens and viral respiratory infections, although adult-onset may occur [5]. The diagnosis of asthma in children may be difficult because episodic respiratory symptoms are common also in those who do not have asthma; hence, a diagnosis can be based on symptom patterns and on a clinical assessment of family history and physical findings, because early allergic sensitization increases the probability that a wheezing child will have asthma [6].

The global prevalence of asthma is difficult to estimate because of different classifications used throughout the world, different methods of assessing asthma in epidemiological studies and the lack of a definitive diagnostic test [7]. Anandan et al. [8] have shown that there is no overall decline in the prevalence of suggestive symptoms of asthma, and in Italy, during the past 20 years, prevalence has raised by 38% [9]. In the framework of the International Study of Asthma and Allergies in Childhood (ISAAC) Project [10], the first SIDRIA (Italian Studies of Respiratory Diseases in Childhood and the Environment) survey in children and adolescents (6–7 and 13–14 years old) was carried out in 10 areas of northern and central Italy [11,12], reporting a lifetime prevalence of 9.1%. In 2002, a second multicentre study including some areas of southern Italy reported a slightly higher prevalence of 9.5% [13]. At present, few data are available on the prevalence of asthma symptoms in some areas of Italy among the childhood population.

The aim of this study was to estimate the prevalence of bronchial asthma and related symptoms in a random sample of children and adolescents aged 0–14 years, living around the industrial area of Termoli, a town in Molise region, Central-South of Italy. The role of several risk factors was also evaluated in order to assess the association with asthma. Furthermore, both indoor and outdoor places frequented and the most common activities carried out by children/adolescents were investigated. Asthma prevalence in the same study area was further estimated by analyzing pediatricians’ databases on drug prescriptions for symptoms control and treatment.

## Methods

### Study design

The survey was carried out between 2008 and 2009 in the main industrial area of the Molise region (Italy). A representative sample of 251 families (n = 665 individuals) was randomly extracted from local register offices. The selection of households was performed according to their residence around the industrial area, including eight neighboring municipalities (Termoli, Campomarino, Guglionesi, Petacciato, Portocannone, San Giacomo degli Schiavoni, San Martino in Pensilis, and Ururi), and to the proportional distribution of general population in each municipality.

Based on similar selection criteria, an additional group of families for each municipality was extracted by systematic sampling with replacement, to ensure a high degree of population representativeness.

For our investigation, the subgroup (n = 95) of children and adolescents aged between 6 months and 14 years was analyzed to assess asthma prevalence. This group represented the 14.3% of the extracted sample (n = 251), in agreement with the distribution of the pediatric population in the selected municipalities in 2008 (14.6%) [14] and 2009 (14.4%) [15]. The study was approved by the Bioethics Committee at the University of Molise, Campobasso, Italy (Protocol number 7469/13).

### Questionnaires and data collection

Asthma symptoms prevalence was measured through the administration of anonymous questionnaires, previously validated in SIDRIA studies, according to the ISAAC Steering Committee methodology [10] with minor modifications. Questionnaires were administered in person to parents of children and adolescents by trained interviewers, who previously obtained informed consent, according to the Italian Legislation, Decree n. 196, 30 June 2003.

Data about Body Mass Index (BMI) calculated according to Cole et al. standard [16], socio-demographic information, current and past asthma symptoms, schooling, lifestyles, children/adolescents’ diseases, parental history of respiratory disorders and education level, were collected.

The exposure to environmental pollutants was also evaluated, including passive smoking, heating systems, molds or dampness at home, and furred pets anytime throughout life. A “confirmed case” was defined on the basis of affirmative responses to the core questionnaire, including the questions “Has your child ever had asthma?”, “Has your child ever had doctor asthma diagnosis?”, “Has your child ever taken any medications or treatment for asthma?”. Furthermore, a “probable case” was identified as an individual who had at least a symptom related to asthma, such as dry cough at night not associated with common cold or chest infections in the past 12 months; wheezing or whistling in the chest during the past 12 months; sleep disturbance due to wheezing; speech limited to few words at a time due to wheezing; audible wheeze during or after physical exercises.

### Weekly individual diary

Parents of children and adolescents were asked to complete the weekly individual diary to investigate the most frequented places, the common activities carried out, and time spent per day in performing some activities. The weekly diary consisted of two tables, the “daily sequence” and “daily activities”. The first table allowed to identify indoor and outdoor places daily frequented by each individual and the most common vehicle used for commuting. The “daily activities” table quantified the time spent performing activities which were classified into rest, sedentary, light, moderate and heavy.

### Drug prescriptions

Information about drug prescriptions for symptom control and treatment were collected by anonymous consultation of pediatricians’ databases to evaluate asthma prevalence in the pediatric population living in the area surrounding the industrial center of Termoli. Data were compared with a lower industrialized area of Campobasso town (Molise region) as control. The inclusion criteria of “confirmed cases” were the use of beta-2 agonist short-acting bronchodilators (salbutamol); recurrent episodes of wheezing (three or more per year); asthma diagnosis according to Global Initiative for Asthma (GINA) international guidelines. Wheezing episodes (<2 per year) associated with occasional respiratory infections were considered as the exclusion criterion.

A total of 1,004 and 920 records of patients living in the area of Termoli and Campobasso were analyzed, respectively.

### Statistical analysis

All the analyses were performed including both “confirmed” and “probable” cases using SPSS release 17.0 (SPSS Inc., Chicago, IL, USA). A p-value < 0.05 was regarded as statistically significant. For symptoms analysis, prevalence data were computed without excluding missing answers, which were therefore counted as negative or “no symptoms”, according to the standard ISAAC practice [12]. Conversely, avoided answers to questions on environmental exposures were properly treated as missing data. Frequency distribution, two-sided Chi-square and Fisher’s Exact test were performed for prevalence comparisons. Multivariate logistic regression was used to calculate Prevalence Odds Ratio (POR), and 95% confidence intervals (CI 95%) to assess the relation between risk factors and asthma cases [17], including the potential confounders.

## Results

In this study, a questionnaire was administered to parents of enrolled (n = 95) children and adolescents. The response rate was of 93.7% (n = 89), because six questionnaires were discarded due to low compliance to their compilation. The mean and the median age of the study population was 7.2 ± 4.4 and 7 years respectively, and a similar distribution by gender (49.4% ♂; 50.6% ♀) was observed, according to the percentage of males and females in 2008–2009 in the general pediatric population in selected municipalities [14,15]. The majority (n = 46, 51.7%) of children/adolescents were from Termoli, followed by Campomarino (n = 11, 12.4%), Petacciato (n = 9, 10.1%) and Ururi (n = 8, 9.0%). A lower representativeness of other municipalities was present, with San Martino in Pensilis accounting for 6.7% (n = 6), followed by Portocannone (5.6%, n = 5), San Giacomo degli Schiavoni (3.4%, n = 3) and Guglionesi (1.1%, n = 1).

Based on the core questionnaire analysis, 22 (24.7%; CI 95% 16.0-34.0) individuals (mean age 5.9 ± 5.0 years) with bronchial asthma were identified, including both “confirmed” and “probable” cases (Table 1). In details, only 7.9% (n = 7/89; CI 95% 2.3-13.5; mean age 7.0 ± 4.6 years; 4♀/3♂) of children/adolescents was defined as “confirmed cases”, most of them (n = 6) living in Termoli. However, six “confirmed cases” (85.7%) did not show asthma symptoms when the questionnaire was administered. Fifteen “probable cases” (16.8%, CI 95% 9.0-24.6, mean age 5.4 ± 3.6 years, 8♀/7♂) were identified, and the 73.3% (n = 11) was from Termoli.

**Table 1:** Description of suggestive symptoms of asthma in children/adolescents (n = 89) enrolled in the study

**Variable**		**No asthma cases**	**Asthma cases**
		**N (%)**	**N (%)**
Ever bronchial asthma*	No	67 (100)	15 (68.2)
	Yes	0 (0)	7 (31.8)
Physical examination*	No	67 (100)	15 (68.2)
	Yes	0 (0)	7 (31.8)
Drug treatment*	No	67 (100)	15 (68.2)
	Yes	0 (0)	7 (31.8)
Wheezing or whistling in the chest at any time in the past	No	62 (92.5)	12 (54.5)
	Yes	5 (7.5)	10 (45.5)
Wheezing or whistling in the chest in the past 12 months	No	61 (91.0)	15 (68.2)
	Yes	6 (9.0)	7 (31.8)
Wheezing or whistling associated to cold or respiratory infections	No	62 (92.5)	21 (95.5)
	Yes	5 (7.5)	1 (4.5)
In the past 12 months wheezing severe enough to limit speech to only one or two words at a time between breaths	No	67 (100)	21 (95.5)
	Yes	0 (0)	1 (4.5)
In the past 12 months a dry cough at night apart from a cough associated with a cold or chest infections	No	65 (98.5)	7 (31.8)
	Yes	1 (1.5)	15 (68.2)
Usually dry cough apart from cough associated with a cold or chest infections	No	57 (87.7)	11 (50.0)
	Yes	8 (12.3)	11 (50.0)
Winter affecting cough frequency	No	61 (91.0)	18 (81.8)
	Yes	6 (9.0)	4 (18.2)
Physical examination for cough	No	59 (88.1)	13 (59.1)
	Yes	8 (11.9)	9 (40.9)
Breathing difficulties following physical exercises	No	59 (90.8)	16 (72.7)
(walking/climbing stairs)	Yes	6 (9.2)	6 (27.3)
Snoring during sleep	No	51 (76.1)	15 (68.2)
	Yes	16 (23.9)	7 (31.8)

Legend: *core questionnaire used to identify the “confirmed cases”.

Asthma symptoms in children/adolescents are described in Table 1. The lifetime prevalence of wheezing or whistling in the chest in the past was reported by 45.5% (n = 10) of asthmatic children/adolescents *vs* 31.8% (n = 7) in the past 12 months, with 1–3 night-time attacks once or twice per week. Among “confirmed” and “probable” cases, 95.5% (n = 21) had wheezing or whistling in the past 12 months not associated with common cold or respiratory infections, 68.2% (n = 15) had nocturnal dry cough in the past 12 months apart from cold or respiratory infections, and 40.9% (n = 9) received a physical examination for cough. Breathing difficulties after physical exercises and snoring during sleep were reported in six (27.3%) and seven (31.8%) cases, respectively.

Within all asthma cases, a similar distribution by gender was observed (54.5% ♀; 45.5% ♂); the majority (n = 16, 72.7%) of children/adolescents were 7 years old or younger. Only 10.0% (2/20) of cases had low (2000–2499 g) birth weight respect to 35.0% (7/20) and 55.0% (11/20) associated to a weight ranged 2500–3499 g and ≥ 3500 g, respectively. A significant difference (p < 0.001) in BMI between asthmatics and non asthma cases was found. In particular, 18.2% (n = 4) and 45.4% (n = 10) of cases were classified as at risk of overweight and obesity, respectively, whereas the 36.4% (n = 8) had lower BMI (Table 2). Asthma was significantly associated with the residence in Termoli (n = 17, 77.3%, p = 0.006), while two cases (9.1%) were from Campomarino, and only one (4.5%) was from Petacciato, Portocannone and San Martino in Pensilis (Table 2). No significant differences were observed regarding the education level of parents, as well as for their employment status and history of respiratory diseases (Table 2).

**Table 2:** Socio-demographic characteristics of children/adolescents (n = 89)

**Variable**		**Non asthma cases**	**Asthma cases**	***p*-value**
		**N (%)**	**N (%)**	
Gender	Female	33 (49.3)	12 (54.5)	0.667^*^
	Male	34 (50.7)	10 (45.5)	
Age	≤ 7 years	34 (50.7)	16 (72.7)	0.071^*^
	> 7 years	33 (49.3)	6 (27.3)	
Birth at term	No	5 (7.5)	1 (4.5)	0.999^**^
	Yes	62 (92.5)	21 (95.5)	
Body Mass Index (BMI)	Other	45 (68.2)	8 (36.4)	< 0.001^*^
	Overweight (25 kg/m^2^)	16 (24.2)	4 (18.2)	
	Obesity (30 kg/m^2^)	5 (7.6)	10 (45.4)	
Overweight/Obesity	No	45 (68.2)	8 (36.4)	0.008^*^
	Yes	21 (31.8)	14 (63.6)	
Residence municipality	Other municipality	38 (56.7)	5 (22.7)	0.006^*^
	Termoli	29 (43.3)	17 (77.3)	
Family size	≤ 3 persons	12 (17.9)	4 (18.2)	0.999^**^
	> 3 persons	55 (82.1)	18 (81.8)	
Paternal education level	≤ Primary school	24 (40.0)	11 (57.9)	0.309^*^
	Secondary school	27 (45.0)	7 (36.8)	
	College/University	9 (15.0)	1 (5.3)	
Maternal education level	≤ Primary school	24 (35.8)	8 (36.4)	0.501^*^
	Secondary school	30 (44.8)	12 (54.5)	
	College/University	13 (19.4)	2 (9.1)	
Paternal employment status	Unemployed	8 (13.3)	4 (21.1)	0.469^**^
	Employed	52 (86.7)	15 (78.9)	
Maternal employment status	Unemployed	4 (6.0)	2 (9.1)	0.634^**^
	Employed	63 (94.0)	20 (90.0)	

Legend: *p-value Chi-square test; **p-value Fisher’s Exact test.

Among enrolled children/adolescents, bronchitis (p = 0.006) and atopic dermatitis (p = 0.005) were significantly related to asthma cases, with a prevalence of 27.3% and 45.5%, respectively.

Information about environmental risk factors exposure was collected. Both confirmed and probable cases were georeferenced based on the familiar residence (data not shown), although clusters nearby the industrial district were not found. Significant differences (p = 0.038) were observed between children/adolescents living in suburban or urban areas, because most of them (19/22, 86.4%) were resident in a rural or suburban zone. Moreover, differences (p = 0.040) were found about the school location, with eleven (61.1%) asthmatics attending school located in an urban area *vs* 38.9% (n = 7) in a suburban zone; most of them (n = 16, 72.7%) were spending up to 5–8 hours/day at school. The presence of an external or internal home heating system was not significant, as well as having air conditioning at home and regular contact with furred pets. Conversely, the self-reported exposure to exhaust gas from industrial processes was significantly (p = 0.024) different between asthmatics and non asthma cases. Both parents current tobacco-smoke exposure and number of cigarettes/day (data not shown) were not related to asthma, whereas maternal history of smoking was slightly significant (p = 0.042). However, no relation with smoking during pregnancy or during the first year of child’s life was found.

Multivariate regression analysis, based on asthma outcome dependent variable, and on the significant independent variables (socio-demographic and indoor/outdoor factors), allowed to estimate Prevalence Odds Ratio (POR) and CI 95% (Table 3), adjusted for the potential confounders (age and gender). In details, only lifetime history of bronchitis, residence of children/adolescents in Termoli town, lifetime atopic dermatitis and high BMI (overweight, obesity) were significantly associated with outcome.

**Table 3:** Variables associated to asthma cases in the multivariate analysis

**Variable**	POR*	CI 95% **	***p*-value**
BMI			
High BMI (overweight, obesity)	8.77	2.40-31.97	0.001
Normal (reference)			
Municipality of residence			
Termoli	8.98	1.17-68.91	0.035
Other municipalities (reference)			
Lifetime history of atopic dermatitis			
Yes	8.78	1.43-54.01	0.019
No (reference)			
Lifetime history of bronchitis			
Yes	17.23	1.89-156.75	0.012
No (reference)			

Legend: *POR, Prevalence Odds Ratio; **CI 95%, Confidence Interval at significance level of 0.05.

Parents of sixty-two (69.7%) individuals filled out the weekly individual diary (average compliance 6.6 ± 1.0 days per week); children/adolescents spent more time in indoor places (mainly at home, followed by school, sport and recreational sites) *vs* outdoor (parks and seaside). The most used vehicle for commuting was the car, followed by bus and train. Moreover, walking, watching television and doing recreational activity or sport were reported as the frequent activities among boys. Conversely, sedentary activities were more common among girls, followed by those light and moderate.

Asthma prevalence in the study area of Termoli was further evaluated by drug prescriptions data analysis, and compared to the control area of Campobasso. Significant differences were found for asthma prevalence (p = 0.005) and gender distribution (p = 0.032) between the two areas. Particularly, among 1,004 pediatrician’s records, the 13.7% (n = 138; CI 95% 11.6-15.8) of children/adolescents (mean age 5.6 ± 3.1 years) was defined as “confirmed cases”. Among these, the 40.6% (n = 56) had an allergic and atopic asthma history, and the 28.3% (n = 39) received prescriptions for chronic symptoms control in the past 12 months. The average number of wheezing episodes per child was 3.7 ± 0.6 per year. In particular, asthma was more prevalent among males (n = 76, 55.1%), and the 81.9% (n = 113) of asthmatic children/adolescents were living in Termoli, followed by Campomarino (7.2%) and Petacciato (6.5%). In the control area of Campobasso, eighty out of 920 (8.6%) children/adolescents were identified as asthmatics, and prevalence was higher (60%, n = 48) among females. Moreover, the 72.5% (n = 58) of cases had an allergic and atopic asthma history, and the 36.2% (n = 29) received prescriptions for symptoms control in the past 12 months. The average number of wheezing attacks per child was 4.1 ± 0.1 per year.

## Discussion

In this study, by using ISAAC questionnaires methodology, including only “confirmed cases”, a prevalence of 7.9% was found which is almost in agreement with previous Italian SIDRIA studies [13], where asthma occurred in the 9.1-9.5% and 9.1-10.4% of children (6–7 years) and adolescents (13–14 years), respectively over 1994–95 and 2002. By drug prescriptions analysis, a higher proportion (13.7%) of diagnosed asthmatic children/adolescents in the same area was observed, highlighting a discrepancy, in agreement with some Authors [18], between questionnaires *vs* pediatrician’s database results; however, these differences were not statistical significant. Indeed, the small number of cases could have affected the discrepancy found in our study. The identification of cases by questionnaire remains a contentious issue [19] respect to anti-asthmatic drug prescriptions methodology, which has the advantage to eliminate selection and recall bias affecting the prevalence estimates in cross-sectional surveys based on self-reported or parental-reported symptoms [20]. However, the use of asthma drugs differs by country (4-26%) and by age, limiting any good comparison between different studies [21].

By including “probable cases”, the overall prevalence of suggestive symptoms of asthma was higher (24.7%) than data referred to Italian childhood population [12,13]. Anyway, to assess the real health condition/status of these children, an accurate medical examination should be conducted.

Over the last years, published data provided a better understanding of the etiology of childhood asthma, improving the significance of socio-demographic and environmental determinants in disease development. Although it is well known that before puberty global prevalence of asthmatic symptoms is higher in boys than girls [22], decreases with age and disappears in adolescence [23], in our study no significant gender-related differences were established when ISAAC questionnaire methodology was followed, and a higher prevalence among girls was found. Based on physician’s prescription data, asthma prevalence was significantly different comparing the industrial area and the control one, and differences between the two areas were confirmed for gender distribution, with prevalence significantly higher in males than in females. To date, asthma has been reported to be 25-70% more common in males compared to females under age 15, while after puberty, the gender differences are reversed [22]. The reduced likelihood of diagnosing asthma in girls could be partly explained by the earlier onset of symptoms and a longer history of wheeze in boys, which gives them more time to be identified as asthmatics. Thus, some under-diagnosing of asthma due to female gender could not be excluded [24]. Recently, sex-specific trends revealed an increased prevalence among females causing a distribution towards the equalization, although male preponderance persists in diagnosed asthma [25].

Our results did not show any association between asthma and low birth weight, which usually represents a predisposing factor for lower lung function and development of disease [26], especially in children/adolescents with birth weight below 3,000 g [27]. Similarly, a high birth weight (> 4,500 g) may be a risk factor for asthma in childhood [28] promoting airways inflammation, but no association could have been evaluated because of low representativeness of this category.

No significant differences comparing children whose mother/father had received less than 13 years of education or more were found, in disagreement with some Authors who reported that a higher maternal and paternal educational level may increase the risk of wheeze/asthma and eczema, respectively [29]. Although some Authors reported that children’s asthma is likely to be more associated with unemployed parents [30], we did not find any relation with parental employment status. Conversely, a significant association with elevated BMI was observed, in agreement with previous SIDRIA and other international studies [26,27] which indicated that overweight and obese children/adolescents are at greater risk of developing asthma. Interestingly, a national survey [31] reported an excess weight in 34.0% of Italian children/adolescents, and an increased prevalence up to 41.3% in Molise region, where the 14.8% and 26.5% are obese or overweight, respectively.

It has been suggested that obesity could precede asthma, increase the clinical severity of disease and reduce quality of life of asthmatics [32]. Possible mechanisms to explain the relation between asthma and high BMI may include airway inflammation, mechanical changes directly related to obesity, changes in airway hyperresponsiveness, physical activity and diet. Moreover, Galassi et al. [13] demonstrated that asthma prevalence in overweight children/adolescents was more frequently reported in areas of southern-Italy, where physical activity is less frequent, and unhealthy diet habits are more common. Lifetime history of both allergic rhinitis and pneumonia was not significantly related to asthma, as well as familiar allergic/respiratory disorders, while researchers have indicated a central role of genetic factors in an increased risk of asthma development [33]. On the contrary, lifetime history of both atopic dermatitis and bronchitis were significant risk factors. These findings are consistent with other studies that reported a higher prevalence of asthma onset in relation to respiratory infections in children [34]. Mechanisms by which respiratory viruses cause asthma exacerbations have been extensively investigated [35]. Bronchitis and asthma are, in fact, considerable respiratory health issues, leading major morbidity and high socio-economic costs [36]. Similarly, atopic dermatitis has been demonstrated as the chronic disease mainly affecting infants and adolescents, with an increased prevalence in childhood asthma and allergic disorders [25,37].

Among the environmental conditions, indoor factors are of particular concern because children and adolescents globally spend more than 80% of their time indoors [2]. To date, the most consistent findings for induction of childhood asthma have been related to tobacco-smoke exposure and maternal smoking during pregnancy [38]. However, our results did not show any relation with both current and prenatal tobacco-smoke exposure, which is generally associated by at least 20% with airway inflammation and an increased incidence of early/persistent wheezing and asthma in children/adolescents [38,39]. Self-reported presence of dampness or visible molds growth in home, associated to wheezing phenotypes and persistent cough, was not related to disease. Furthermore, no significant relation with furred pets exposure was observed, whose role in allergic diseases and asthma development is still controversial [40].

Exposure to outdoor pollutants was evaluated by parent self-reported assessment, and being resident in an area exposed to exhaust gas from industrial processes was significantly different in asthma cases compared to non asthmatics, but further insights are needed to clearly define any hypothesis. The relation between asthma and residence zone was investigated, but relevant differences were found only for school and residing location in an urban or suburban area. A significant association with outcome was found for asthmatic children/adolescents living in the municipality of Termoli that may be subjected to higher environmental pressure due to industrial/manufacturing activities and presence of toll road, state highway, railroad, and seaport which may cause air pollution from motor vehicle traffic and increase asthma induction, according to other studies [19,41,42].

We acknowledge some limitations of the study, such as the use of parental-reported symptoms, whose accuracy was not confirmed, and sample size for a definitive evaluation of asthma risk factors.

## Conclusions

Although our data indicated a prevalence concordance with previous national studies in pediatric population, results did not confirm any hypothesis related to environmental industrial factors present in the study area. In fact, only past history of atopic dermatitis and bronchitis, as well as high BMI, were strongly associated to the outcome. This study provides a first report on asthma prevalence in Molise region, contributing to complete the frame of disease epidemiology in Italy in meta-analysis or systematic review procedures, and to identify interesting relationships in the context of multiple observational studies. Indeed further insights are needed to improve the understanding of the epidemiological situation in the survey area, and the contributions of environmental stimuli in asthma development.

## Competing interests

The authors declare that they have no competing interests.

## Authors’ contributions

GR conceived of the study, participated in its design and coordination, and helped to analyze the data and draft the manuscript; MT performed the descriptive and the statistical analyses of data, and helped in writing the manuscript; MLS participated in the design of the study, in questionnaires predisposition and analysis, and helped to draft the manuscript; GdL carried out the data collection from pediatricians’ databases, and helped in writing questionnaires; AB participated in the design of the study, and analyzed and evaluated the clinical data. All authors read and approved the final manuscript.

## Pre-publication history

The pre-publication history for this paper can be accessed here:

http://www.biomedcentral.com/1471-2458/13/1038/prepub
